# Kinetic Profile of Urine Metabolites after Acute Intake of a Phenolic Compounds-Rich Juice of Juçara (*Euterpe edulis* Mart.) and Antioxidant Capacity in Serum and Erythrocytes: A Human Study

**DOI:** 10.3390/ijms24119555

**Published:** 2023-05-31

**Authors:** Alyne Lizane Cardoso, Luciane de Lira Teixeira, Neuza Mariko Aymoto Hassimotto, Sheyla de Liz Baptista, Cândice Laís Knöner Copetti, Debora Kurrler Rieger, Francilene Gracieli Kunradi Vieira, Gustavo Amadeu Micke, Luciano Vitali, Maria Alice Altenburg de Assis, Mayara Schulz, Roseane Fett, Edson Luiz da Silva, Patricia Faria Di Pietro

**Affiliations:** 1 Graduate Program in Nutrition, Department of Nutrition, Federal University of Santa Catarina, Florianopolis 88040-900, SC, Brazil; 2 Department of Food Science and Experimental Nutrition, University of São Paulo, São Paulo 05508-900, SP, Brazil; 3 Department of Chemistry, Federal University of Santa Catarina, Florianopolis 88040-900, SC, Brazil; 4 Department of Food Science and Technology, Federal University of Santa Catarina, Florianopolis 88034-001, SC, Brazil; 5 Graduate Program in Nutrition, Department of Clinical Analysis, Federal University of Santa Catarina, Florianopolis 88040-900, SC, Brazil

**Keywords:** *Euterpe edulis*, phenolic compounds, anthocyanins, urine metabolites, antioxidant activity

## Abstract

The juçara palm tree produces a small spherical and black–purple fruit similar to açaí. It is rich in phenolic compounds, especially anthocyanins. A clinical trial evaluated the absorption and excretion of the main bioactive compounds in urine and the antioxidant capacity in serum and erythrocytes of 10 healthy subjects after juçara juice intake. Blood samples were collected before (0.0 h) and 0.5 h, 1 h, 2 h, and 4 h after a single dose (400 mL) of juçara juice, while urine was collected at baseline and 0–3 and 3–6 h after juice intake. Seven phenolic acids and conjugated phenolic acids were identified in urine deriving from the degradation of anthocyanins: protocatechuic acid, vanillic acid, vanillic acid glucuronide, hippuric acid, hydroxybenzoic acid, hydroxyphenylacetic acid, and ferulic acid derivative. In addition, kaempferol glucuronide was also found in urine as a metabolite of the parent compound in juçara juice. Juçara juice caused a decrease in the total oxidant status of serum after 0.5 h in comparison to baseline values (*p* < 0.05) and increased the phenolic acid metabolites excretion. This study shows the relationship between the production of metabolites of juçara juice and the total antioxidant status in human serum, indicating evidence of its antioxidant capacity.

## 1. Introduction

Intake of fruits and vegetables has been inversely associated with the development of chronic diseases, such as cardiovascular diseases, cancer, and neurodegenerative diseases [1,2,3,4]. The *Euterpe edulis* palm tree, popularly known as juçara, is a tropical palm tree native to the Brazilian Atlantic Forest [5,6] spread especially in Brazil’s Southeast and South regions [7]. This palm tree produces small spherical and black–purple fruits, very similar in sensory and nutritional characteristics to açaí (*Euterpe oleracea* Mart), a Brazilian Amazonian fruit that presents several bioactive molecules with antioxidant properties, that can potentially improve the vital functions of erythrocytes and, therefore, body homeostasis [8], with both berries having a diameter of 1 to 1.5 cm [9]. Juçara pulp has been inappropriately called açaí to give it commercial relevance despite it belonging to a different palm tree species [7]. However, according to a previous study, juçara fruit has a better nutritional composition than açaí, with higher contents of linolenic and linoleic acids, potassium, calcium, magnesium, ascorbic acid, phenolic compounds, anthocyanins, and antioxidant capacity [9]. Furthermore, the excessive extraction of the heart of palm leads to its extinction. At the same time, the consumption of its fruits stimulates the cultivation of the plant and preserves it since the seeds allow the propagation of new palm trees. Moreover, from the environmental perspective, juçara fruit is an important food source for many birds, such as toucans, guans, jacutingas, curassows, thrushes, and various mammals, such as rodents, marsupials, primates, and bats [10]. Therefore, juçara fruit has been considered a sustainable alternative with nutritional and economic relevance, with 150 to 200 tons produced annually in Brazil [11]. Studies have proposed the potential health benefits of juçara fruit associated with the antioxidant effects reported in vitro [12], in vivo through animal models [13], and via clinical trials [14,15,16]. Furthermore, other aspects such as triglycerides [13] and low-density-lipoprotein-cholesterol (LDL-c) [13,17] reduction, low-density-lipoprotein-cholesterol (HDL-c) increase [15], glycemic response improvement [18], and prebiotic effects [19] have been demonstrated. 

Recently, our research group compiled the biological effects of juçara fruit with potential benefits to humans [15,16,17,18,19,20]. The health benefits of juçara fruit might be related to its phenolic compounds profile, especially flavonoids (such as anthocyanins) and phenolic acids [7,12,21]. For example, anthocyanins have been associated with several health effects, such as antioxidant [14,15,16], anti-inflammatory [22], and anticarcinogenic properties [23].

According to Sies [24], oxidative stress is “*an imbalance between oxidants and antioxidants in favour of the oxidants, leading to a disruption of redox signaling and control and/or molecular damage*”. In this context, the potential beneficial effects of juçara fruit due to its high antioxidant content may neutralize oxidative stress.

The bioavailability of anthocyanins is considered very low, with the maximum plasma concentration estimated in nanomoles or nanograms, and urinary excretion being less than 0.1% of intake [25,26]. In addition, anthocyanins are extensively metabolized into phenolic acids, which may also be responsible for the health effects of phenolic compounds-rich food intake [25,27]. The metabolism of anthocyanins into phenolic acids might be due to the chemical degradation by gut microbiota. Therefore, considering total phenolic acids as their end products, the bioavailability of anthocyanins increases to around 12.0% [28,29].

As knowledge about the metabolism of bioactive compounds is still scarce, the study of intake of phenolic compounds-rich sources, such as juçara fruit, can improve the understanding of its impact on the risk factors for chronic diseases, helping the design of future clinical trials.

Previously, the intake of juçara juice by healthy subjects [14,15,16] showed relevant attenuation in the serum marker of oxidative damage and increased serum antioxidant capacity. In the present study, we expanded the knowledge about juçara juice, evaluating its absorption and excretion of anthocyanin metabolites in urine and the antioxidant capacity in serum and erythrocytes of healthy individuals.

## 2. Results

### 2.1. Chemical Composition and Antioxidant Capacity of Juçara Juice

Table 1 shows the chemical composition and the main phenolic compounds in the juçara juice. Phenolic acids, anthocyanins, flavanonols, flavonols, stilbenoids, flavan-3-ols, phenolic aldehyde, and flavone were detected in the juçara juice.

The phenolic acids identified were gallic (GA), protocatechuic (PCA), *p*-coumaric (*p*-CA), vanillic (VA), ferulic (FA), chlorogenic (5-O-caffeoylquinic acid, 5-CQA), caffeic (CA), and syringic acids (SA). PCA, VA, FA, and CA were the most abundant. The total anthocyanin content in 400 mL juçara juice drank by volunteers was 659 mg. The two main anthocyanins were cyanidin 3-*O*-glucoside and cyanidin 3-*O*-rutinoside. The antioxidant capacity (2,2-diphenyl-1-picrylhydrazyl (DPPH) and ferric reducing antioxidant potential (FRAP) assays) was also measured (Table 1). The juçara juice presented a FRAP value of 1664 µmol ascorbic acid equivalent/400 mL and a DPPH of 3740 mg ascorbic acid equivalent/400 mL.

### 2.2. Phenolic Acid Identification and Pharmacokinetic Parameters

Table 2 shows the flavonoid and phenolic acid metabolites detected in the urine samples of healthy subjects after the juçara juice intake. 

Table 3 shows the pharmacokinetic parameters accessed (maximum concentration, Cmax; and area under the curve (AUC)) at 6 h after the juçara juice intake for the quantified metabolites (PCA, VA, VA glucuronide, hydroxybenzoic acid (HBA), hippuric acid (HA), hydroxyphenylacetic acid (HPA), kaempferol glucuronide). Trace amounts of ferulic acid (FA) derivative were also detected.

**Figure 1:**
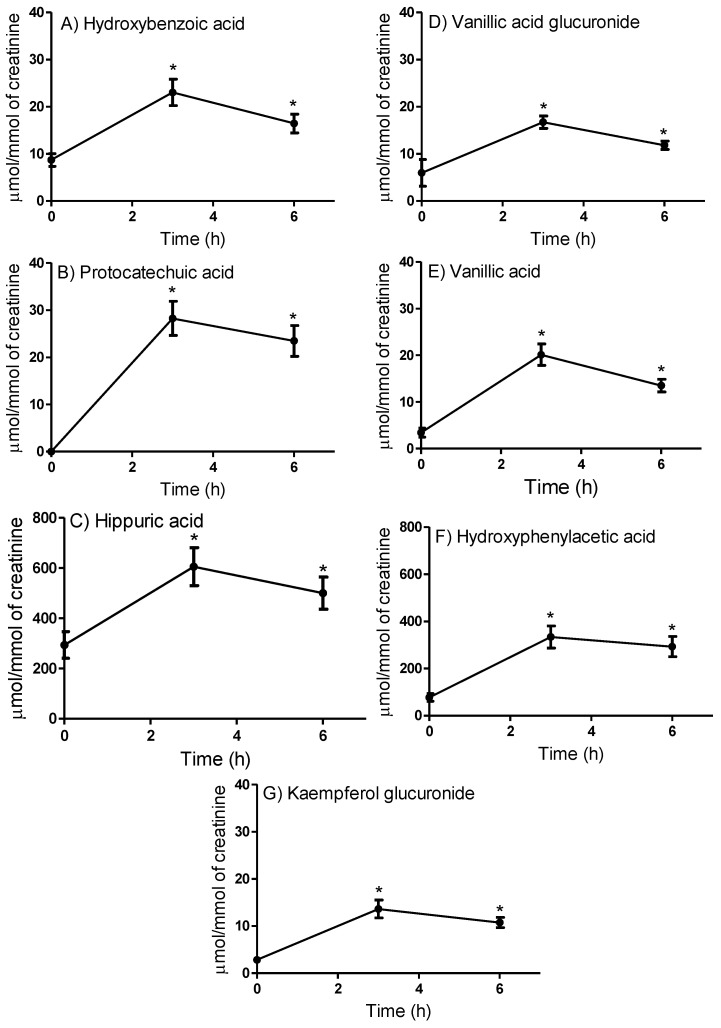
Concentration of phenolic compounds quantified in the urine of ten subjects after consumption of the juçara juice: hydroxybenzoic acid (**A**), protocatechuic acid (**B**), hippuric acid (**C**), vanillic acid glucuronide (**D**), vanillic acid (**E**), hydroxyphenylacetic acid (**F**), and kaempferol glucuronide (**G**). No significant differences were detected between the tests carried out 0–3 h and 3–6 h after the intake. Data expressed as the means and standard error of the mean (SEM). Note: * *p*-values < 0.05 compared to the baseline (n = 8–10); one-way ANOVA and Tukey’s post hoc test.

All phenolic compounds had their maximum concentration in the 0–3 h range after the juçara juice intake (Figure 1). Furthermore, as shown in Figure 1, the mean concentration of urine metabolites accessed 0–3 and 3–6 h after the juçara juice intake for the ten subjects showed no significant variation between them. However, they were significantly higher than at baseline, before the juçara juice intake (*p* < 0.05). The metabolites identified in urine in the highest concentrations after the juçara juice intake were HA and HPA.

### 2.3. Antioxidant Capacity in Serum and Erythrocytes

**Figure 2:**
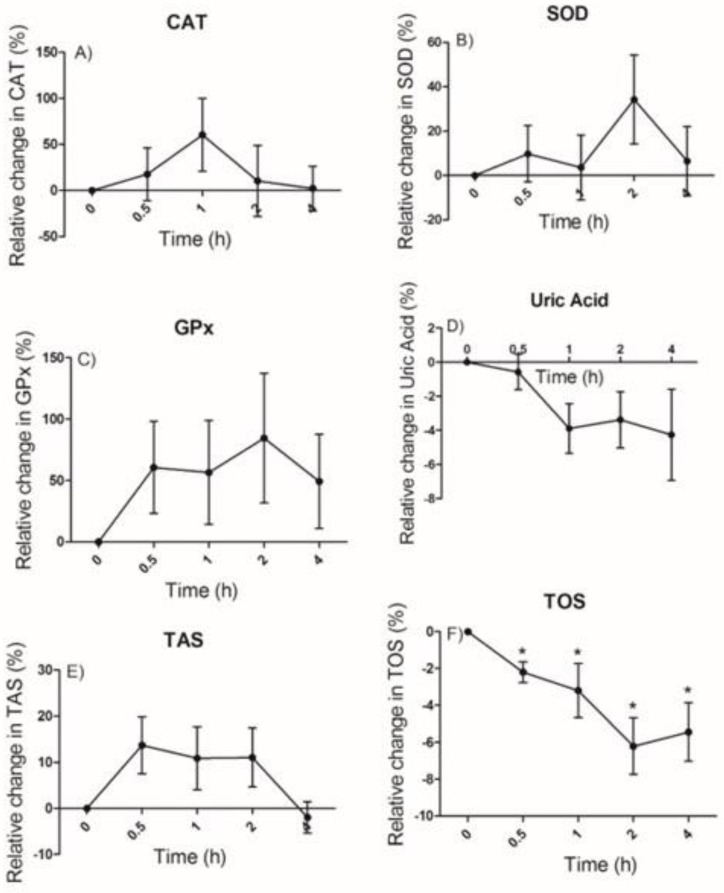
Relative changes in the serum and erythrocyte antioxidant parameters catalase (CAT) (**A**), superoxide dismutase (SOD) (**B**), glutathione peroxidase (GPx) (**C**), uric acid (**D**), total antioxidant status (TAS) (**E**), total oxidant status (TOS) (**F**) after the juçara juice consumption. Data are expressed as the means and SEM. Note: * *p*-values < 0.05 compared to the baseline (n = 8–10); one-way ANOVA and Tukey’s test.

Figure 2 shows the relative changes in the parameters catalase (CAT), superoxide dismutase (SOD), and glutathione peroxidase (GPx) activities, uric acid, total antioxidant status (TAS), and total oxidant status (TOS).

There were no significant differences in the TAS, uric acid, and CAT, SOD, and GPx activities. However, the juçara juice intake decreased the TOS values at 0.5 h, 1 h, and 2 h, compared to the baseline (*p* < 0.05). Although not statistically significant, CAT, SOD, and GPx showed activity peaks between 1 h and 2 h after the juçara juice intake that coincided with the increased excretion of phenolic acids identified in the urine for the collection time range 0–3 h.

## 3. Discussion

To the best of our knowledge, this is the first study to demonstrate the metabolism of phenolic compounds of juçara juice in humans. Phenolic acids, anthocyanins, flavanonols, flavonols, stilbenoids, flavan-3-ols, and flavones were detected in juçara juice. Secondary metabolites of plants can be divided into flavonoids and non-flavonoids [3]. Among the non-flavonoids are phenolic acids and stilbenes, while flavonoids are represented by anthocyanins, flavanols, flavonols, flavones, flavanones, and isoflavones [30]. PCA, VA, GA, *p*-CA, FA, 3-CQA, CA, and syringic acid were the second metabolites identified in the juçara juice. PCA, VA, FA, and CA were the most abundant. All phenolic acids were also identified in other studies [21,31,32]. Further investigations have described the rutin and quercetin flavonoids in juçara fruit extract [12,21,32,33]. Herein, relevant contents of kaempferol and aromadendrin were also found in the juçara juice, confirming data from Schulz et al. [31]. Other compounds identified in the juçara juice in this study, such as taxifolin, resveratrol, catechin, and epicatechin, have already been reported by other authors [31,34]. It is important to note that the phenolic compounds profile in juçara fruit may vary according to its maturation, cultivation region, and climatic conditions [7,12,33]. 

In this study, the two main anthocyanins observed in the juçara juice were cyanidin 3-*O*-glucoside and cyanidin 3-*O*-rutinoside, corroborating other studies [32,33,35]. The antioxidant capacity of juçara fruit has been reported by several studies, with the parameters of DPPH [15,16,31,32,33] and FRAP [15,16,17] being the most studied. The DPPH value was higher than those reported in other studies. De Liz et al. [15] and Copetti et al. [16] described 603.5 and 156.0 mg ascorbic acid equivalent/100 mL juice, respectively. In contrast, these authors found FRAP values of 2095.0 [15] and 453.0 [16] µmol ascorbic acid equivalent/100 mL juice. Compared to açaí juice, the DPPH value in the present study was fourfold higher than that mentioned by De Liz et al. [15], while the FRAP value was higher in açaí juice (570.0 µmol ascorbic acid equivalent/100 mL). 

The present study measured anthocyanins and their metabolites only in urine. Nonetheless, the presence of anthocyanins in plasma is recognized to be very low, with only trace amounts reaching the bloodstream and being excreted with urine (<0.1% in human studies) [36,37]. Such results indicate that anthocyanins have an intensive metabolism before being eliminated with urine. In addition, the absorption and excretion of anthocyanins depend on several factors that can influence their metabolism, such as food matrix [38], interindividual variability [39], and degradation by intestinal microflora [28]. Therefore, the primary anthocyanin metabolites excreted in urine were phenolic acids, especially HA and HPA. The gut microbiota might metabolize the non-absorbed anthocyanins firstly undergoing glycosidic linkages and further degradation to phenolic acids, which may be absorbed by the colon epithelium [3,37].

Although we did not measure metabolites in plasma, HA was identified as one of the major metabolites of cyanidin-3-glucoside in urine, reaching a maximum concentration of 6058.0 µmol/mmol creatinine. According to the literature, we may speculate that HA is formed via PCA metabolism, which is the product of spontaneous degradation of cyanidin-3-glucoside in the small intestine and circulation [28,40]. Additionally, the human liver might be responsible for the further breakdown of anthocyanin aglycones to PCA [41]. In the present study, VA, the methylated metabolite of PCA, and VA glucuronide were also measured in the urine after the juçara juice intake, indicating possible excretion via PCA metabolites. The phenolic acids, such as PCA and FA derivatives identified in the urine may also be partially absorbed from the parent compounds present in juçara juice. Kaempferol glucuronide also appeared in urine after absorption of the parent compound identified in the juçara juice. This might also be the case, at least partially, for VA and its glucuronidated phase II metabolite. Metabolites of a typical anthocyanin incubated with CACO-2 cell were PCA, syringic acid, VA, HA, and GA [42]. It is important to note that phenolic acids such as PCA, syringic acid, VA, HA, GA, *p*-CA, FA, 3-CQA, and CA have also been identified in juçara fruit [12,21,31,33], which may contribute to their direct absorption and excretion after juçara juice intake.

HPA appeared in urine as the second major metabolite of cyanidin-3-glucoside, reaching a maximum concentration of 3340.9 µmol/mmol creatinine. Such a metabolite could be produced from the breakdown of anthocyanin aglycone to form dihydrophenylacetic acid, with further dehydroxylation to HPA [41]. HA is normally found in urine samples, in the samples of all volunteers at baseline. However, as expected, an increase in concentration was observed after the juice intake. The baseline values can be different between the volunteers. The normal amount of HA in urine samples can vary according to alcohol and vegetable intake, age, gender, exposure to toluene, and smoking habit [43]. Its value in toluene-free healthy volunteers is about 126.36–245.13 µmol/mmol creatinine [43,44], which is similar to the average value of the baseline samples (before the juice intake) obtained in our study (around 243.06 µmol/mmol creatinine). Thus, we selected healthy volunteers, and the subjects were instructed to follow a low antioxidant diet for 48 h to avoid further variations in the data.

The juçara juice decreased the TOS values at 0.5 h, 1 h, and 2 h after the juice intake. These findings corroborate the previously published study, which showed positive effects after an acute intake of juçara juice on lipid peroxidation in 11 healthy subjects [14].

Antioxidants play a role against free nitrogen and oxygen radicals formed in tissues and biological fluids [45]. In the present study, different parameters were evaluated to accurately assess the antioxidant capacity in serum and erythrocytes. The SOD, CAT, and GPx enzymes work in parallel [46], and the assessment of all three must be relevant to analyze the antioxidant status defense system [20]. It has been proposed that the health effects of anthocyanin-rich foods come mostly from anthocyanin metabolites such as phenolic acids rather than anthocyanin. These benefits include increased serum antioxidant capacity [25,27], also observed in the present study. Furthermore, Fernandes et al. [47] suggested that the benefits of anthocyanin-rich fruit might be related to the constant release of phenolic compounds through the gut and blood circulation.

According to a recent review, the effects of intervention with juçara on antioxidant enzymes remain controversial [20]. Some experimental studies showed decreased SOD and CAT activities [13,48]. In contrast, other studies reported an increase in the CAT activity in an animal model [49] and humans [19] and an enhanced GPx activity in humans [14]. Anthocyanins and/or their metabolites can stabilize reactive species and affect the endogenous antioxidant capacity [25].

As investigated in the present study, other crossover clinical trials also examined the effects of juçara juice intake on healthy subjects [15,16]. In a clinical trial, the authors showed a decrease in the oxidative stress index and fatigue immediately post-exercise and an increase in reduced glutathione (GSH) 1 h later. An increase in total phenols and uric acid over time was also observed. There were no significant differences in the activities of antioxidant enzymes GPx, SOD, and CAT [16]. De Liz et al. [15], using a four-week clinical trial, demonstrated that juçara juice elevated the TAS levels and the CAT and GPx enzyme activities and decreased the oxidative stress index. Moreover, juçara juice increased the HDL-c levels. As a result, these studies suggested that juçara juice intake could reinforce the antioxidant defense system and alleviate oxidative stress.

Our study and the three trials that assessed the antioxidant effects of açaí (*Euterpe oleracea*) in healthy subjects showed a different response pattern according to the intervention model [50,51,52]. For example, in acute trials, a significant increase in antioxidant capacity was observed at 2 h after açaí juice intake [50,51], similarly to the results obtained in the present study. In addition to the elevation of the total antioxidant capacity [52,53], an increase in the antioxidant protection of erythrocytes and CAT activity and a decrease in the reactive species generation [53] were observed in the medium- to long-term interventions. Thus, the duration of intervention and study design might influence interventions using juçara and açaí juices. 

As strengths of this study, it should be mentioned that this was the first study to evaluate the absorption, excretion, and pharmacokinetic parameters of the main phenolic acids and anthocyanins of juçara juice in the urine of healthy subjects after acute intake. On the other hand, the primary limitations concern the absence of urine sample collection at timepoints after 6 h, losing some metabolite detection. The lack of measuring metabolites in plasma has also been considered a limitation of this study. However, it is well-known that the maximum concentration of anthocyanins in humans occurs at 0.5–4 h after intake. The average urinary excretion is between 0.03% and 4% of the ingested dose, eliminated between 1.5 and 3 h [51]. In addition, several factors can interfere with the assessment of anthocyanin absorption, such as interindividual variability, composition of the gut microbiota, and measurement of metabolites.

## 4. Materials and Methods

### 4.1. Chemicals

We purchased 2,2′-azino-bis-(3-ethylbenzothiazoline-6-sulfonic acid) (ABTS), acetone, acetonitrile, citric acid, DPPH, ferrous-ion-*o*-dianisidine, Folin–Ciocalteu phenol reagent, formic acid, gallic acid, hydrochloric acid, methanol, β-nicotinamide adenine dinucleotide 2-phosphate reduced tetrasodium salt (NADPH), ultrapure phenolic standards, 4-amino-3-hydrazino-5-mercapto-1,2,4-triazole (Purpald), sodium acetate, superoxide dismutase determination kit, 2,4,6-tris(2-pyridyl)-s-triazine (TPTZ), and 6-hydroxy-2,5,7,8-tetramethylchromane-2-carboxylic acid (Trolox) from Sigma-Aldrich Chemical Co. (St. Louis, MO, USA). Ascorbic acid, boric acid, ferric chloride, ferrous sulfate, hydrogen peroxide, potassium chloride, sodium carbonate, sodium hydroxide, and sulfuric acid were obtained from Vetec (Rio de Janeiro, RJ, Brazil). Cyanidin-3-*O*-glucoside, delphinidin-3-*O*-glucoside, delphinidin, quercetin-3-*O*-glucoside, 3-O-caffeoyl-quinic acid, and myricetin were purchased from Extrasynthese (Genay, France). HA, PCA, VA, isovanillic acid (iVA), ellagic acid, FA, and kaempferolwere purchased from Sigma-Aldrich (St Louis, MO, USA). The kit to analyze urine creatinine was purchased from Labtest Diagnóstica S.A. (Lagoa Santa, MG, Brazil).

#### 4.1.1. Chemical Characterization of the Juice

The juçara juice was produced by a specialized company (Duas Rodas) located in southern Brazil from fruits harvested in the municipality of Garuva (latitude: 26°01′36″ S, longitude: 48°51′18″ W, altitude: 25 m), Santa Catarina, Brazil, on November 2015. The samples followed processing and pasteurization standardization according to the information provided by the company. Aliquots with 5 L juçara juice in each packing bottle were available and later divided into the amounts administered in this study.

Chemical parameters were determined by the following methods recommended by the Association of Official Analytical Chemists (AOAC, 2005): moisture (925.09) was determined by drying the sample at 105 °C until obtaining constant weight and ash by muffle incineration (AOAC, 940.26); crude protein was determined by the Kjeldahl method (920.87), and the total nitrogen content was calculated using a conversion factor of 6.25; total lipids were determined according to the Soxhlet extraction method (920.85).

#### 4.1.2. Antioxidant Capacity

The antioxidant capacity of juçara juice was evaluated by the DPPH radical scavenging and FRAP methods.

The juçara juice samples were sequentially extracted according to the method proposed by Rufino et al. [54] with some modifications. Briefly, 4 mL of the samples were extracted with 10 mL methanol for 60 min in an ultrasonic bath (Unique, model USC-1400, São Paulo, SP, Brazil) and centrifuged at 5300× *g* for 15 min. The supernatant volume was elevated to 25 mL using a volumetric flask. The juçara juice was dried (freeze dryer LT 1000, Terroni, São Paulo-SP, Brazil). The residue was extracted with 4 mL acetone/water (7:3 *v*/*v*) in an ultrasonic bath for 60 min and centrifuged for 15 min at 5300× *g*. The supernatant was transferred to the same 25 mL flask and completed with deionized water. The DPPH radical-scavenging activity was based on the method proposed by Brand-Williams [55]. The working solution was obtained by diluting the DPPH solution with methanol to obtain an absorbance of around 0.800 (±0.02) at 515 nm. A 2.9 mL aliquot of this solution was mixed with 100 μL of the extracts at varying concentrations. The solution was shaken in tubes and incubated in the dark for 30 min at room temperature. The absorbance was then measured at 515 nm. Inhibition of the DPPH free radicals, in percent terms, was calculated as follows:

% inhibition = [1 − (sample absorbance t = 30 min/control absorbance t = 0 min)] × 100

A standard curve was used, and the results were expressed as mg ascorbic acid equivalent/400 mL juçara juice. The FRAP assay was performed according to the method described by Benzie and Strain [56]. The extract (200 μL) and 200 μL FeCl_3_ (3 mmol L^−1^ in 5 mol L^−1^ citric acid) were mixed in a tube and incubated for 30 min in a water bath at 37 °C. The TPTZ solution (3.6 mL) was then added and mixed. After exactly 10 min, the absorbance was read at a wavelength of 620 nm. The results were expressed as mg ascorbic acid equivalent/400 mL juçara juice.

#### 4.1.3. HPLC-DAD Analysis

Phenolic compounds and anthocyanins were determined by diluting the juçara juice in ultrapure water (1:2 *w*/*w*), extracted with acetonitrile, and centrifuged at 8000× *g* for 10 min at 4 °C. The supernatant was used to perform the analysis. The samples were extracted twice. The anthocyanins were identified and quantified in a high-performance liquid chromatography (HPLC) system (Shimadzu LC-20AT, Kyoto, Japan) equipped with a diode array detector (DAD; Shimadzu, SPD-M20A, Kyoto, Japan) and a reversed-phase C18 column (150 mm × 4.6 mm ID) with a particle size of 5 µm (Supelco, Nucleosil, Bellefonte, PA, USA). The gradient elution in liquid chromatography was composed of mobile phases A (0.1% formic acid) and B (0.1% formic acid in 95% methanol: 5% water) as follows: 0–2 min, 95% A; 2–7 min, 95% B; 7–20 min, 95% B. The injection volume was 20 µL for all samples, and detection was performed at 520 nm. The identified peaks were confirmed using an authentic standard of cyanidin 3-*O*-glucoside and cyaniding 3-*O*-rutinoside (Extrasynthese, Genay, Lyon, France) dissolved in acidified methanol (0.1% HCl). Quantification was performed using calibration curves (7–10 concentrations: 0.01–2 μg/L), and the data were expressed as mg/400 mL juçara juice.

#### 4.1.4. LC–ESI–MS/MS Conditions

The phenolic compound contents (mainly phenolic acids) in the juçara juice were also determined by liquid chromatography-electrospray ionization–tandem mass spectrometry (LC–ESI–MS/MS) on a 1200 HPLC system (Agilent Technologies, Waldbronn, Germany) coupled to a hybrid quadrupole linear ion trap mass spectrometer QTRAP^®^ 3200 (Applied Biosystems/MDS Sciex, Toronto, ON, Canada) equipped with a TurboIonSpray^TM^ source (electrospray, ESI). Separation was performed with a Synergi column (4.6 µm particle size, 150 mm, 2.0 mm). The gradient elution in liquid chromatography was composed of mobile phases A (95% methanol) and B (0.1% formic acid) as follows: 0–5 min, 10% A; 5–7 min, 90% A; 7–10 min, 90% A; 10–17 min, 10% A. The gradient elution for anthocyanin identification was the same as in Section 4.1.3. The flow rate was 250 µL/min, the analyses were carried out in the negative and positive ion modes, and the capillary needle was maintained at 4500 V. The MS/MS parameters were a curtain of hot nitrogen gas at 10 psi; a temperature of 400 °C; gas at 1.45 psi; gas at 2.45 psi; and a medium collisionally activated dissociation (CAD) gas. Quantification was based on the calibration curves using standards (95–98% purity), and the concentrations were expressed as mg per 400 mL of the juice. Phenolic standards were dissolved in methanol or methanol acidified as described in Section 4.1.3. 

### 4.2. Human Study Design

The sample size was based on the previous studies that evaluated the effect of a single intake of açaí pulp on human plasma antioxidant activity [50,51] and the anthocyanins’ pharmacokinetics of açaí juice and pulp intake [51]. This study was registered at https://ensaiosclinicos.gov.br (RBR-9cb3n9) and approved by the Ethics Committee on Human Research of Universidade Federal de Santa Catarina (registration No. 33131414.2.0000.0121). All the subjects gave their written informed consent.

#### 4.2.1. Participants

Ten subjects were selected, six women and four men, aged between 23 and 30 years, with the body mass index (BMI) between 18.5 and 29.9 kg/m^2^.

Study volunteers were selected according to the following inclusion criteria: no clinical conditions such as cardiovascular, endocrine, gastrointestinal, renal, or hepatic diseases; no visible or known infections or inflammatory processes within three months of the study; no use of medicines or dietary supplements, no smoking, no regular alcohol drinking. The other criteria were BMI < 30 kg/m^2^ and no gastric intolerance or complications related to the juçara juice intake. The subjects were instructed to follow a low antioxidant diet for 48 h according to a previously published research protocol [14]. The subjects were advised to avoid vegetable and fruit sources of anthocyanins or their derivatives, such as açai, blackberries, blueberries, cherries, red and purple grapes, strawberries, and green and black tea, two days before the study. A prospective food record was carried out to evaluate the compliance with the low antioxidant diet 48 h before the intervention. The subjects were also instructed not to practice intense physical activity and not to deprive themselves of sleeping before the outset of the study protocol. Blood and urine samples were collected after 12 h fasting. Subsequently, the participants drank 400 mL of the juçara juice within 5 min. Blood (30, 60, 120, and 360 min) and urine (0–3, 3–6 h) were collected again. The volunteers continued fasting during the 6 h of the sample collection time. The study protocol was based on evidence that anthocyanins appear to be rapidly absorbed and then eliminated within 4 h [26,36,47,57]. Adverse events were not observed during the study protocol. Blood oxidative stress biomarkers were assessed, and urine metabolites of phenolic compounds were measured. 

#### 4.2.2. Blood Collection and Biochemical Analysis

Blood was collected using a vacuum system (Vacutainer-BD, São Paulo, SP, Brazil) in tubes with or without ethylenediaminetetraacetic acid (EDTA). The tubes were centrifuged for plasma and serum isolation (1000× *g*, 15 min, at 4 °C). Total blood was hemolyzed and antioxidant enzymes SOD, GPx, and CAT were measured as previously described [13]. The samples were maintained in an ultra-freezer at −80 °C until analyses. The TAS) was evaluated following Erel’s automated method [58], which assesses the ability of antioxidants to neutralize the ABTS cation, originating a chromophore with the maximum absorption at 660 nm. The results were expressed as millimoles of Trolox equivalent per liter (mmol TE/L). The TOS was assessed according to Erel [59] and expressed in μmol H_2_O_2_ equiv./L. The CAT enzyme activity was measured with Purpald at 240 nm [60]. The SOD enzyme activity was measured with a kit (Sigma-Aldrich^®^). The GPx activity was assessed by monitoring the oxidation of NADPH in the presence of hydrogen peroxide as described by Wendel [61]. 

### 4.3. Metabolites in Urine

#### 4.3.1. Sample Preparation and Extraction

Phenolic compounds were extracted in urine using Waters OASIS HLB μElution plates (Milford, MA, USA), which were preconditioned with 250 μL methanol and 250 μL 0.2% acetic acid. After urine collection, the samples were immediately acidified with trifluoracetic acid. Aliquots of the samples were applied to the μ Columns of the plates and the phenolic compounds were eluted with methanol acidified with 0.2% acetic acid. The eluate was dried using CentriVap Benchtop Vacuum Concentrators (Labconco, Kansas City, MO, USA), reconstituted with methanol acidified with 5% acetic acid, and filtered with a 0.43 μm PTEF filter (Millipore, Bedford, MA, USA).

#### 4.3.2. HPLC Analysis

Metabolites were quantified using a 1260 Infinity Quaternary LC System liquid chromatograph (Agilent Technologies, Santa Clara, CA, USA) coupled with a quaternary pump, an autosampler, and a DAD. The column used was a 5 Prodigy ODS3 column (250 × 4.60 mm) (Phenomenex Cheshire, UK) with a 1 mL/min flow rate and a column temperature of 25 °C. The mobile phase consisted of two solvents: (A) 0.5% formic acid in water and (B) 0.5% formic acid in acetonitrile. The gradient used was 10% (B) at the beginning, 10% at 5 min, 20% at 15 min, 25% at 25 min, 35% at 33 min, 50% at 38 min, 90% at 43–44 min and 10% at 45 min. Anthocyanins and other phenolic compounds were detected by monitoring elution at 525 and 270 nm, respectively. Quantification was based on the calibration curves of the PCA, VA, iVA, HA, HBA, and HPA standards. The concentrations were expressed as μmol/mmol creatinine for urine samples, and the elimination values were expressed in μmol. Urinary creatinine was evaluated using a Labtest kit (Labtest Diagnóstica S.A., Lagoa Santa, Minas Gerais, Brazil). The area under the curve (AUC) was calculated using the trapezoidal rule (data expressed as μmol/h), and the maximum concentration (Cmax) was calculated using the Origin 6.0 software (OriginLab Corporation, Northampton, MA, USA) and expressed as the mean and standard error of the mean (SEM). The samples collected from 0 to 3 h (after the juçara juice intake) were indicated as 3 h urine and the samples collected from 3 to 6 h were designated as 6 h urine to indicate the period of excretion. The standards’ purity varied from 95 to 98%.

#### 4.3.3. LC–ESI–MS/MS Analysis

Phenolic compounds’ metabolites were identified using a Prominence Liquid Chromatograph (Shimadzu, Japan) coupled to an ion trap Esquires-LC mass spectrometer (Bruker Daltonics, Billerica, MA, USA) with an electrospray ionization (ESI) interface. The chromatography condition was the same as that used for metabolite quantification by HPLC-DAD. After passing through the DAD detector, the flow rate was changed to 0.2 mL/min to inject the samples into the mass spectrometer. ESI was left in the positive mode for anthocyanins and in the negative mode for the other compounds. The mass spectrometer condition was collision energy of 4500 and 4000 V for the positive and negative modes, respectively, and capillary temperature of 275 °C. Scanning *m*/*z* was 100–1500. The compounds were identified by comparison with the retention times of authentic standards when possible and by the similarity of the absorption spectrum, mass spectral characteristics, and comparison with the literature.

### 4.4. Statistical Analysis

Data normality was verified by means of the Shapiro–Wilk test. All the data were normally distributed and are presented as the means ± standard deviation (SD) or the SEM. Relative changes in the oxidative stress biomarkers in the post-consumption serum and erythrocytes compared to the baseline levels were evaluated by means of one-way ANOVA and Tukey’s post hoc test. Differences in metabolite levels in urine collected before and 3 h and 6 h after the juçara juice intake were analyzed using one-way ANOVA and Tukey’s post hoc test. All the data were analyzed using Stata version 11.0 for Windows (Stata Corporation, College Station, TX, USA), considering a significance level of less than 5% (*p* < 0.05).

## 5. Conclusions

This study provides the first evidence of the excretion of phenolic compounds after juçara juice intake by healthy subjects. The results showed that juçara juice intake leads to the production of metabolites (PCA, HA, and HPAs) most likely derived from anthocyanins. In addition, protection against oxidative stress was modulated by juçara juice intake, as observed by the decrease in TOS levels. Further pharmacokinetic and metabolomic studies are recommended to approach the complete excretion of phenolic compounds after intake of juçara juice through a longer intervention period.

## Figures and Tables

**Table 1:** Chemical and phenolic compounds composition and in vitro antioxidant capacity of the serving size of juçara juice (400 mL).

Components	Mean ± SD
Dry matter (g)	25.84 ± 1.00
Ash (mg)	0.80 ± 0.04
Protein (g)	2.04 ± 0.12
Lipids (ethereal extract) (g)	7.00 ± 0.08
**Phenolic acids (mg/400 mL)**	
Gallic acid	0.08 ± 0.01
3,4-Dihydroxybenzoic acid (protocatechuic acid)	1.13 ± 0.07
*p*-Coumaric acid	0.10 ± 0.01
Vanillic acid	0.46 ± 0.00
Ferulic acid	0.22 ± 0.02
Chlorogenic acid	0.04 ± 0.01
Caffeic acid	0.41 ± 0.03
Syringic acid	0.08 ± 0.01
**Flavanonols**	
Taxifolin	0.36 ± 0.01
Aromadendrin	1.22 ± 0.02
**Flavonols**	
Rutin	0.01 ± 0.00
Quercetin	0.04 ± 0.01
Isoquercetin	0.03 ± 0.00
Kaempferol	1.35 ± 0.10
**Stilbene**	
Resveratrol	0.06 ± 0.00
**Phenolic aldehyde**	
Vanillin	0.02 ± 0.01
**Flavanols**	
Catechin	0.35 ± 0.02
Epicatechin	0.13 ± 0.04
**Flavone**	
Apigenin	0.33 ± 0.03
**Anthocyanins**	
Cyanidin 3-*O*-glucoside	462.04 ± 27.31
Cyanidin 3-*O*-rutinoside	197.04 ± 31.93
**Antioxidant capacity**	
DPPH ^a^	3739.64 ± 316.96
FRAP ^b^	1663.28 ± 249.92

^a^ 2,2-diphenyl-1-picrylhydrazyl (DPPH) values are expressed in mg ascorbic acid equivalent and standard deviation (SD). ^b^ Ferric reducing antioxidant potential (FRAP) values are expressed in µmol ascorbic acid equivalent and SD.

**Table 2:** Phenolic acids and metabolites identified in the urine samples of healthy subjects by HPLC–ESI–MS/MS following the juçara juice intake.

Identified Compounds (Negative Mode)	RT (min)	[M − H+]^−^ (*m/z*)	MS2 (*m/z*)
Protocatechuic acid ^a^	7.6	153	109
Vanillic acid glucuronide	8.4	343	167
Hippuric acid ^a^	13.6	178	134
Vanillic acid ^a^	14.2	167	–
Hydroxybenzoic acid ^a^	12.0	137	–
Hydroxyphenylacetic acid ^a^	12.8	151	–
Kaempferol glucuronide	14.3	463	287
Ferulic acid derivate	23.6	193/175	193/175

^a^ Compound identity was confirmed with commercial standards. RT, retention time; MS2, two tandem mass spectrometry.In the present study, parent anthocyanins were not detected in urine, only their metabolites. The primary anthocyanin metabolites excreted were phenolic acids. Seven phenolic acids and one flavonol were identified in urine (Table 3).

**Table 3:** Pharmacokinetic parameters of phenolic metabolites detected in the urine of 10 healthy subjects following ingestion of the juçara juice.

Metabolites	*C*_max_ (µmol/mmol Creatinine)	AUC (µmol/h)
Protocatechuic acid	28.26 ± 3.62	282.89 ± 49.31
Vanillic acid	20.13 ± 2.27	191.42 ± 25.98
Vanillic acid glucuronide	16.74 ± 1.33	164.38 ± 14.98
Hydroxybenzoic acid	23.04 ± 2.80	231.09 ± 36.75
Hippuric acid	605.80 ± 75.74	6483.34 ± 1159.82
Hydroxyphenylacetic acid	334.09 ± 46.79	3527.78 ± 657.52
Kaempferol glucuronide	13.62 ± 1.89	138.05 ± 20.58

Data expressed as the means and SEM; AUC: area under the curve (0–6 h); *C*_max_: maximum concentration; n = 9–10.

## Data Availability

The data presented in this study are available on request from the corresponding author.
